# Precocity

**Published:** 1861-03

**Authors:** 


**Precocity.**

The following remarkable case of precocious menstruation is reported to the *Journal of Materia Medica* by J. B. Somers M. D., of Seaville, N. J. He says:

"I feel it not only a duty but a pleasure, Mr. Editor, to place within your hands an account (however imperfect it may be in detail) of a case that has recently been under my charge. It is that of a girl now nine years of age, who commenced to menstruate in the spring of '59, being then at the age of seven and a half years.

"The case having afforded me no little interest, I doubt not that it will also be satisfactory to some of your readers. She commenced to menstruate in May of the above year, having completed her seventh year the previons October, and continued thus to do until the following spring, when she became anæmic and dejected, and menstrual flow suddenly ceasing. Upon the least exercise her respiration was greatly hurried, and ever likely to swoon upon the least excitement. Her pulse became enfeebled and appetite ceased. In this condition I found her, surrounded with friends filled with alarm, which, by the way, did not tend to lessen the excitability of her mind. She was placed upon the use of chalybeate preparations, stimulating food, and, with moderate out door exercise, began immediately to amend. As amendment advanced, more vigorous exercise was demanded and ere she had completed her ninth year she was as healthy and robust as any of her playmates. Her catamenia was restored as her general health improved, and that it may hereafter be well with her, she is "out on the ocean sailing." Although young, she has the appearance of age, but in intellect as well as in years she is no more than a child."

